# Supplementary figures and images for: Using contact network dynamics to implement efficient interventions against pathogen spread in hospital settings: A modelling study
Source: PLoS Med. 2024 Jul 30;21(7):e1004433. doi: 10.1371/journal.pmed.1004433 (PMC11341093; doi:10.1371/journal.pmed.1004433)

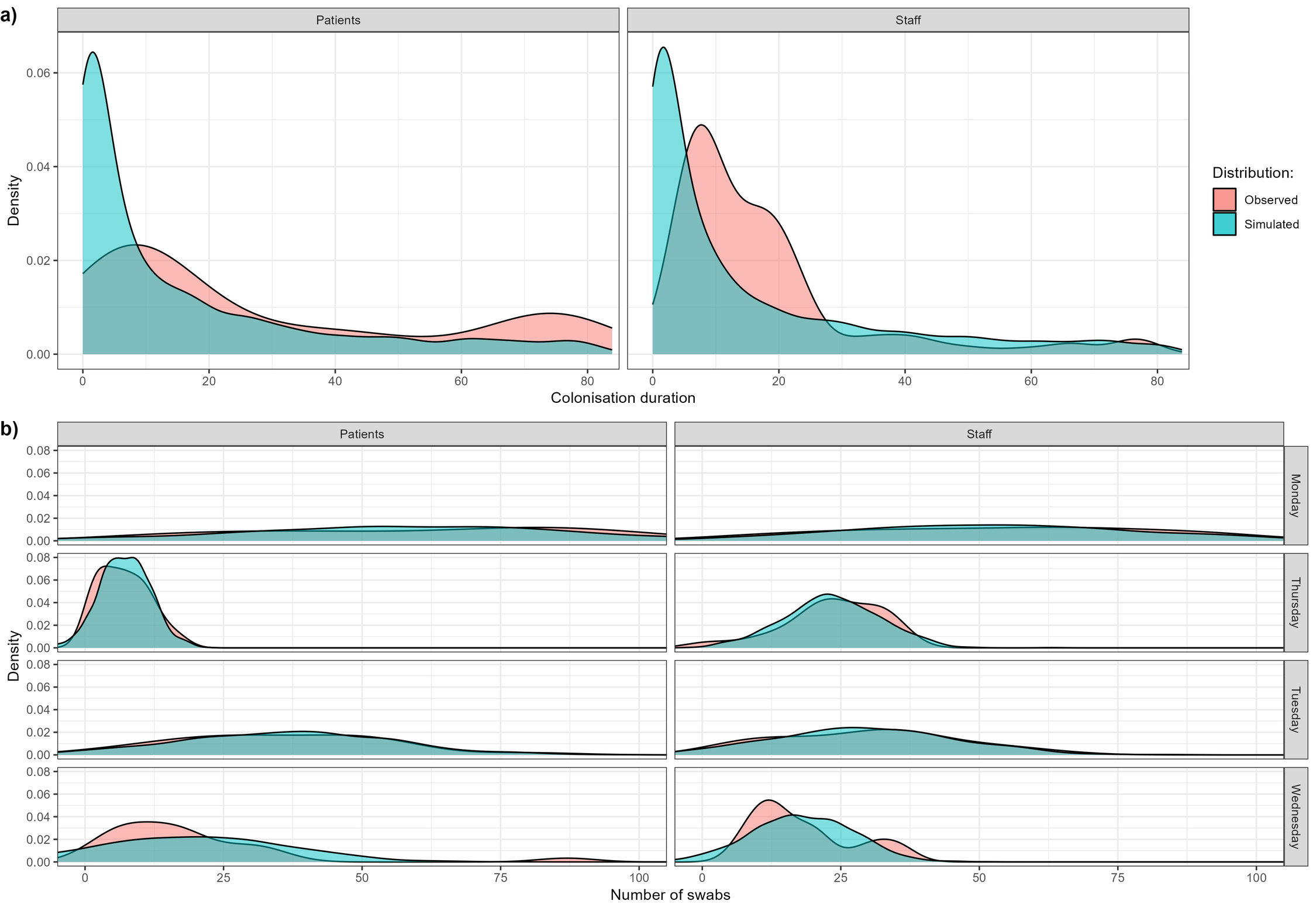

Supplement: S1 Fig — The observed distributions in pink are the smoothed densities of observations in the i-Bird study data, while the simulated distributions in blue are the smoothed densities generated by Lognormal (for colonisation durations, a)) or Normal (for number of swabs, b)) distributions informed by the mean and variance of the data. (TIF) [file pmed.1004433.s001.tif]

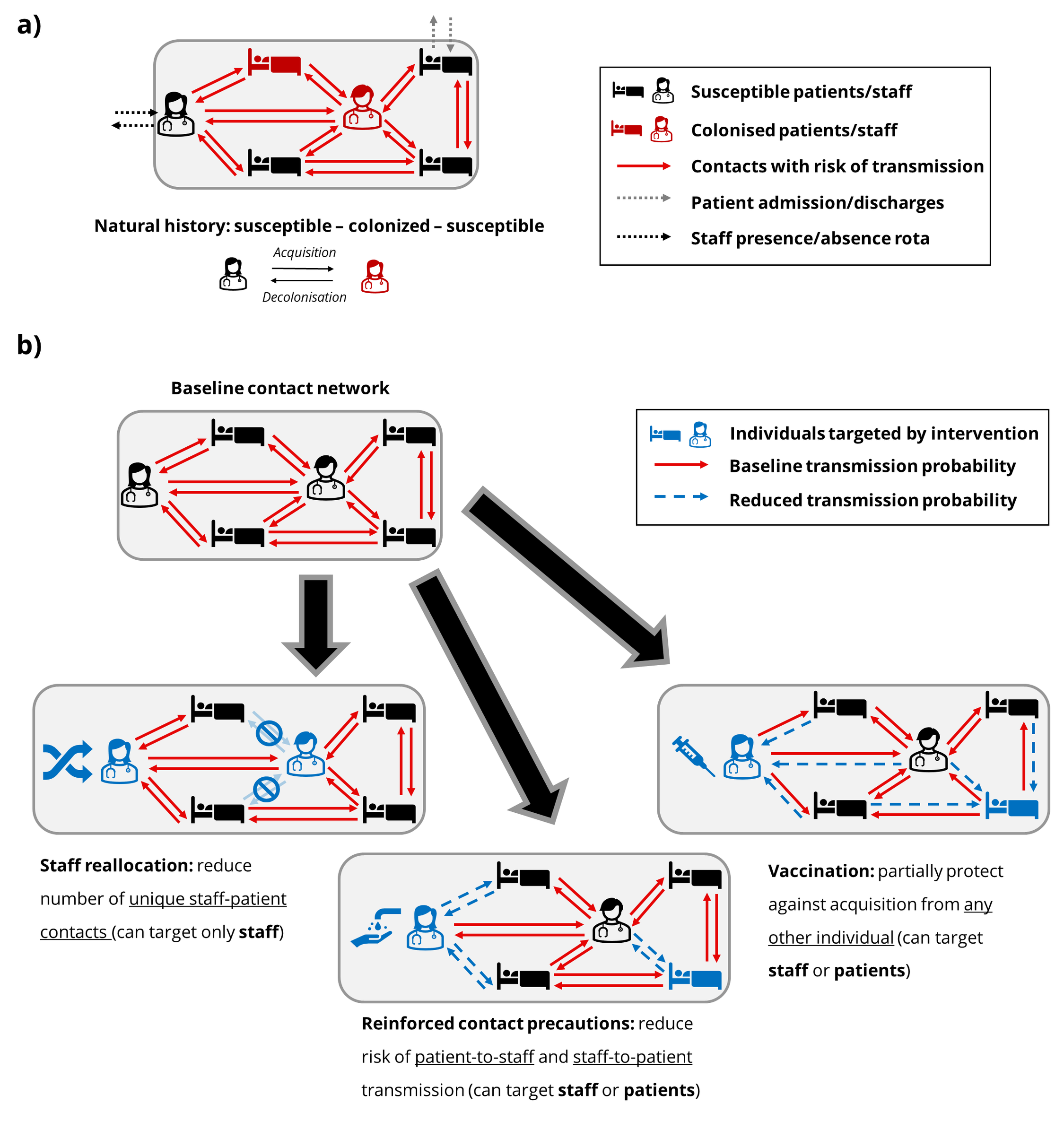

Supplement: S2 Fig — (a) Baseline model description and disease natural history. (b) Mode of action of the 3 different interventions examined in the model. (TIF) [file pmed.1004433.s002.tif]

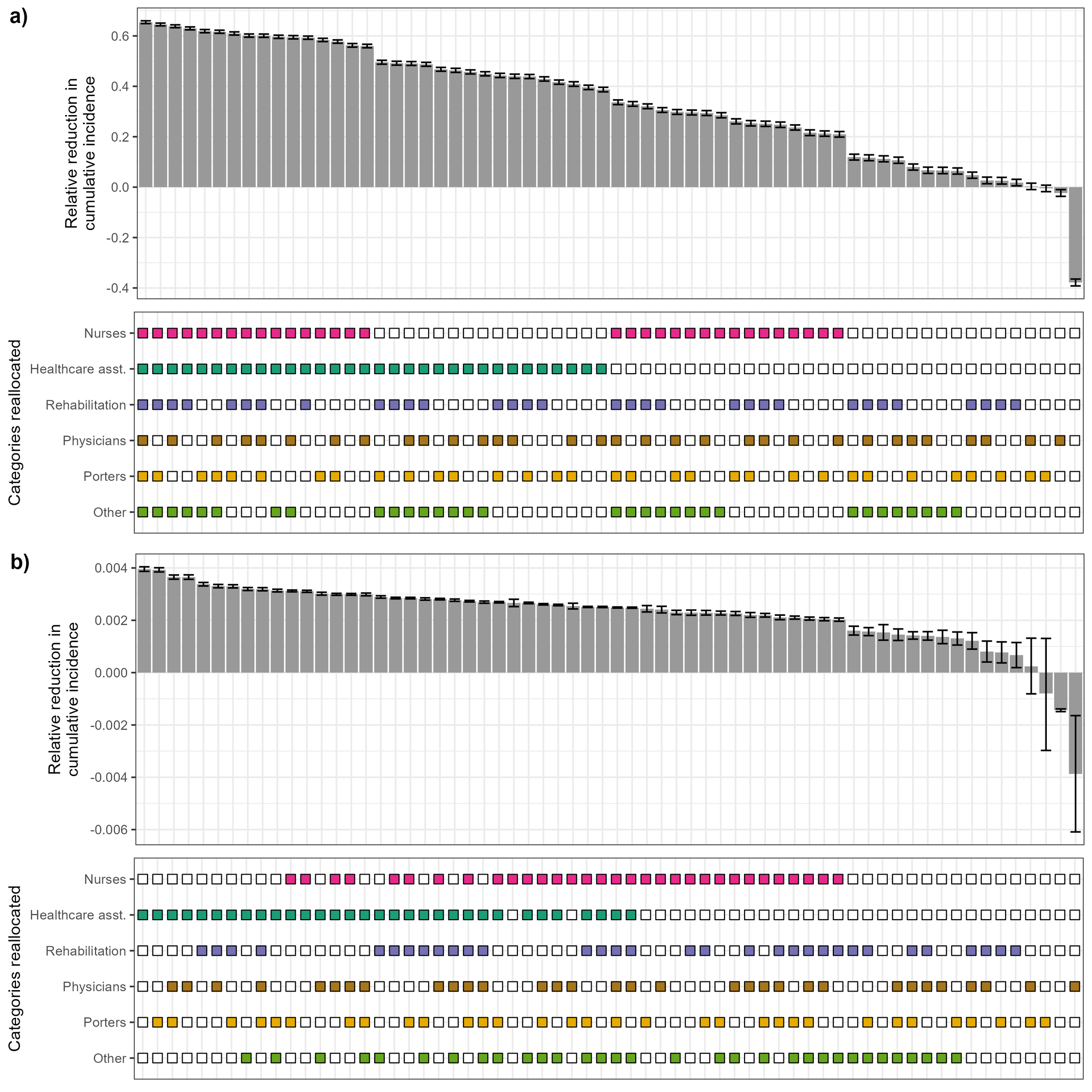

Supplement: S3 Fig — Top: Each bar depicts, for a given scenario, the median relative reduction between 500 model simulations with no intervention, and 500 simulations with staff reallocation, along with the 95% confidence interval. A negative reduction indicates that the intervention led to an increase in cumulative incidence. Bottom: In each scenario, staff categories coloured are those reallocated. In scenario 64, the contact network is random. In each plot, the scenarios are ranked from most to least effective. (TIF) [file pmed.1004433.s003.tif]

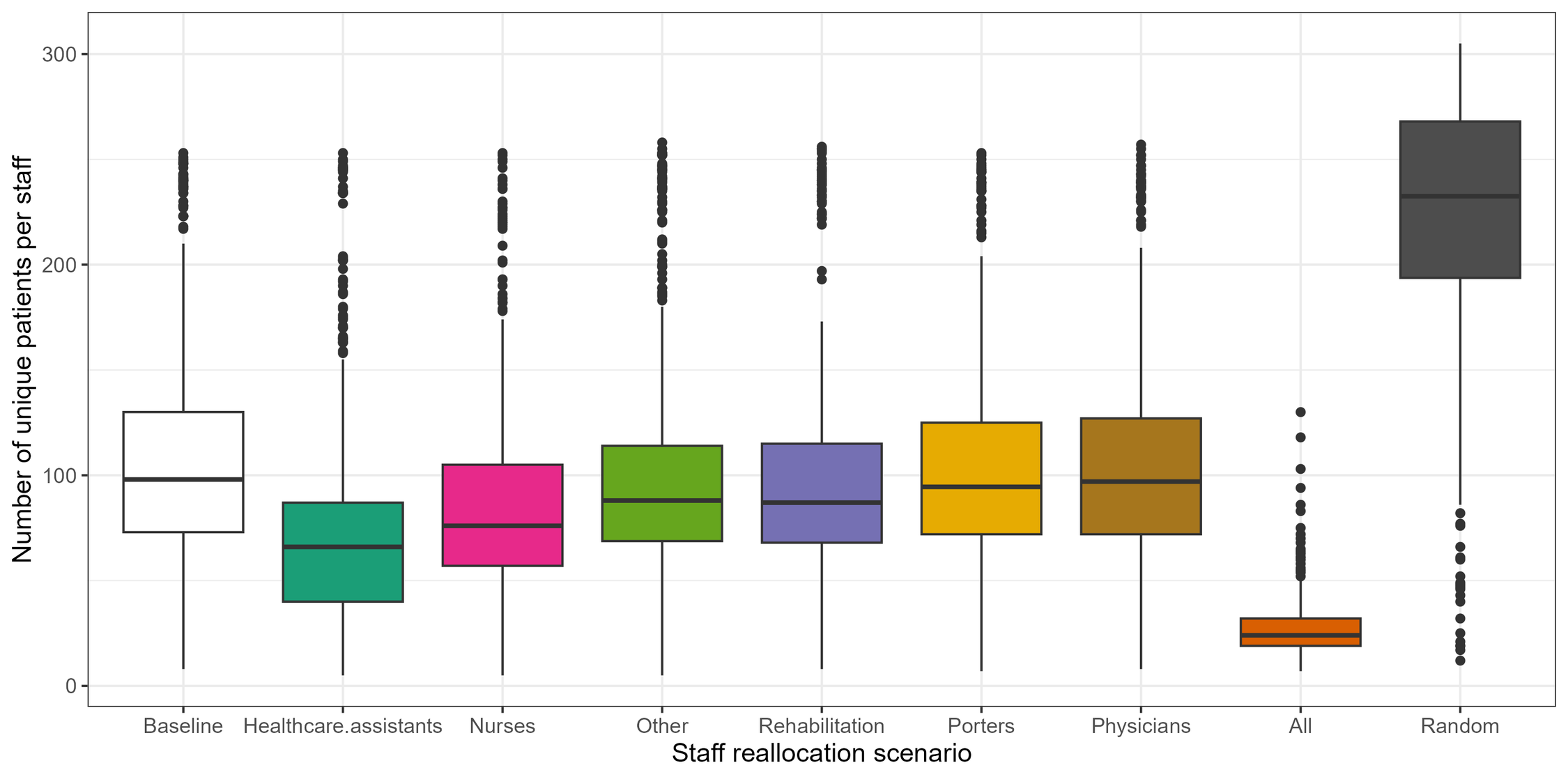

Supplement: S4 Fig — (TIF) [file pmed.1004433.s004.tif]

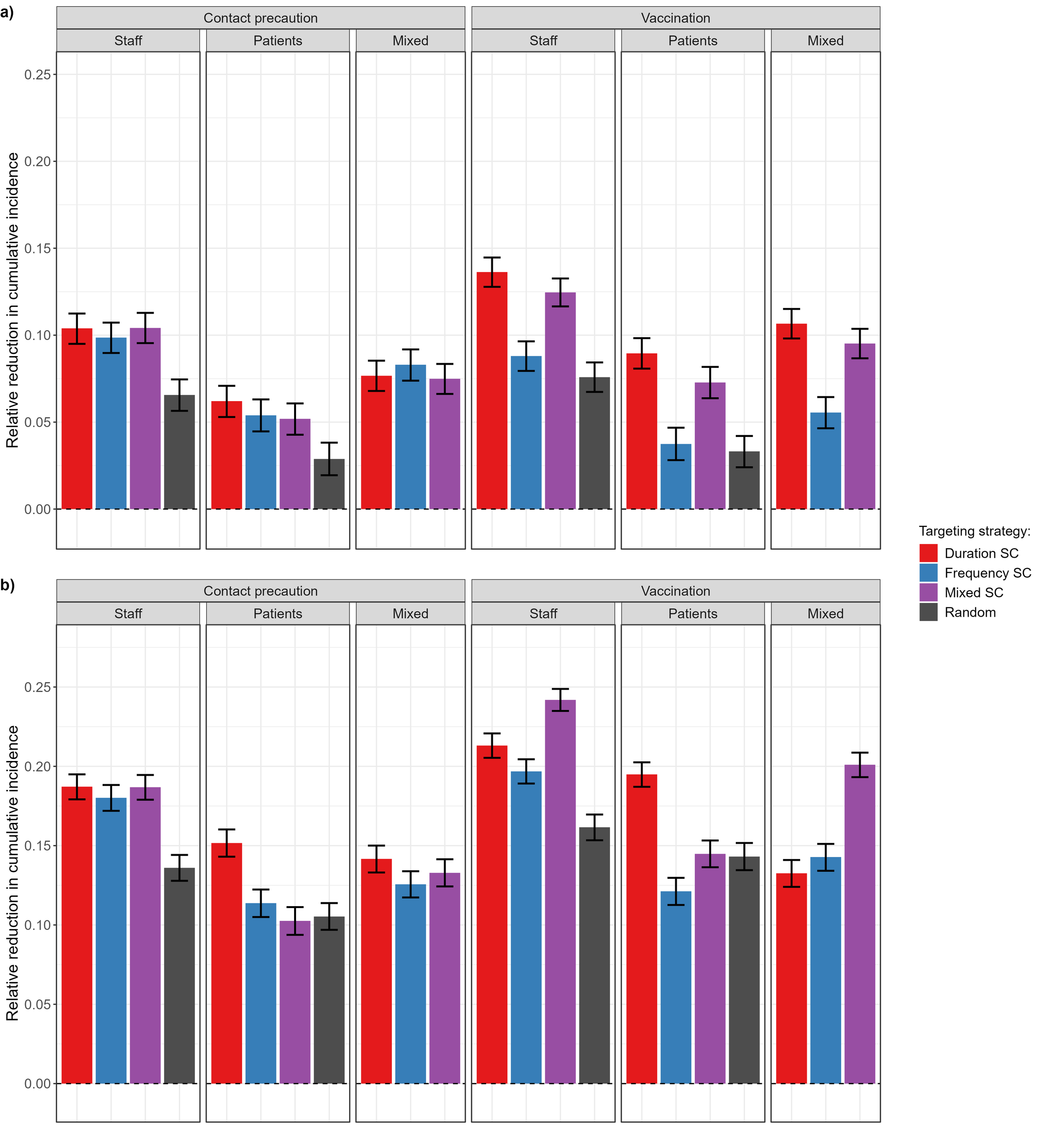

Supplement: S5 Fig — For each strategy, the bar indicates the median relative reduction in cumulative incidence, with 95% confidence interval, obtained for 500 simulations. (TIF) [file pmed.1004433.s005.tif]

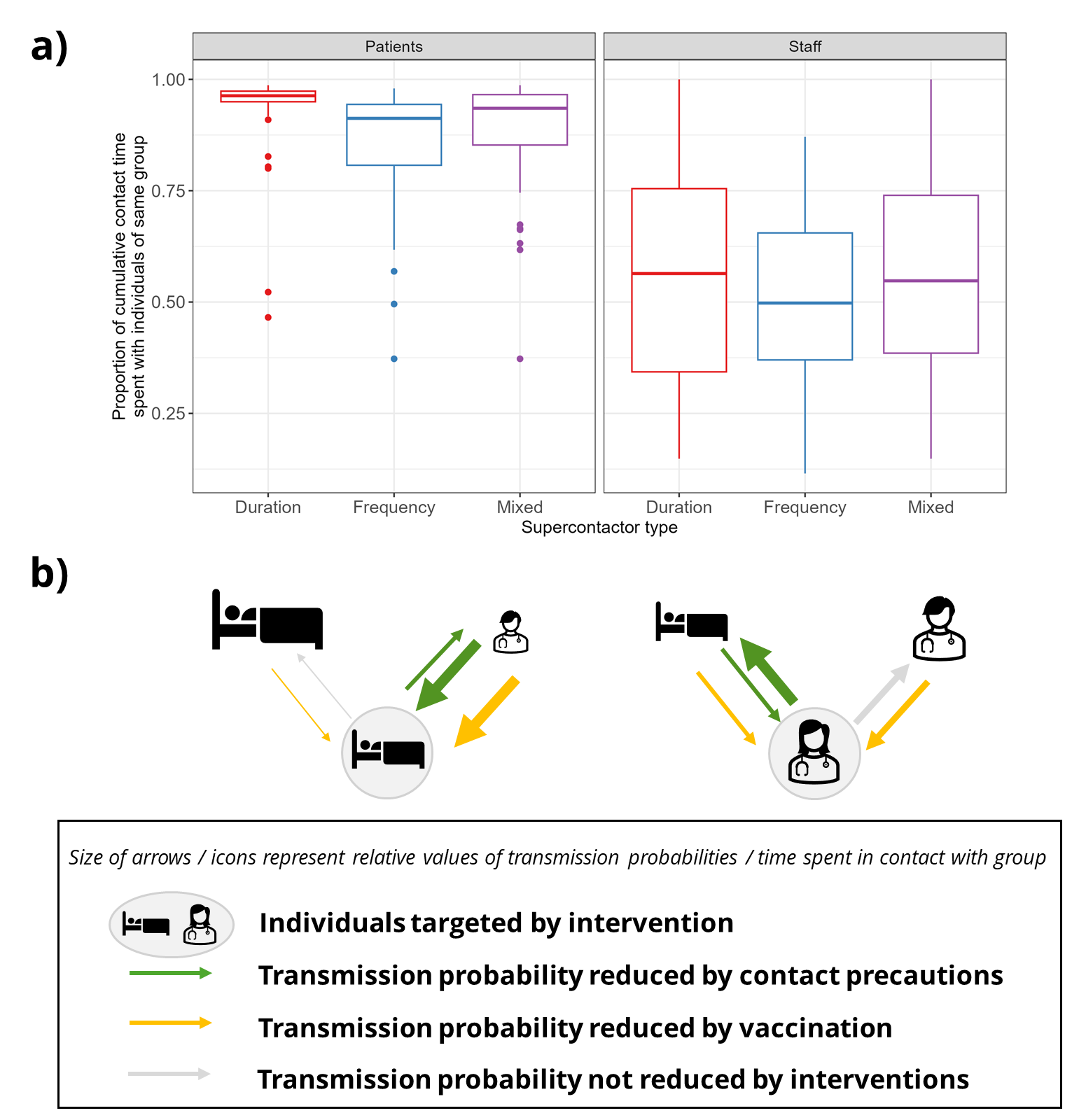

Supplement: S6 Fig — (a) Most of the cumulative contact time of patient supercontactors is with other patients, while staff supercontactors spend approximately the same amount of time in contact with either patients or staff. (b) Contact precautions and vaccination do not reduce the same transmission probabilities. For example, for patients, vaccination may be more effective than contact precautions since it reduces the largest per-contact transmission probability (staff-to-patient) and reduces the probability for the most dominant type of contact (patient-to-patient). (TIF) [file pmed.1004433.s006.tif]

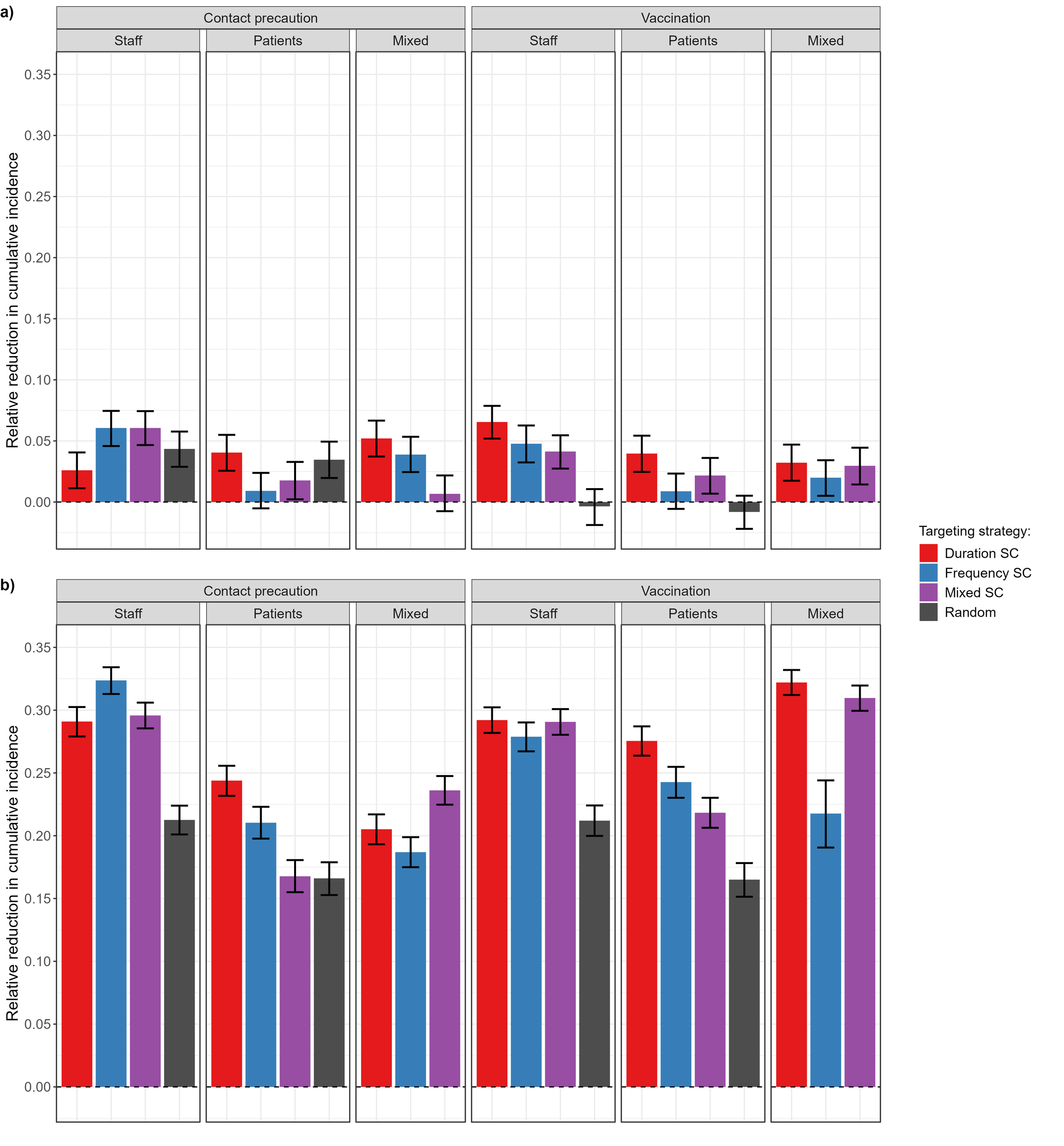

Supplement: S7 Fig — For each strategy, the bar indicates the median relative reduction in cumulative incidence, with 95% confidence interval, obtained for 500 simulations. (TIF) [file pmed.1004433.s007.tif]

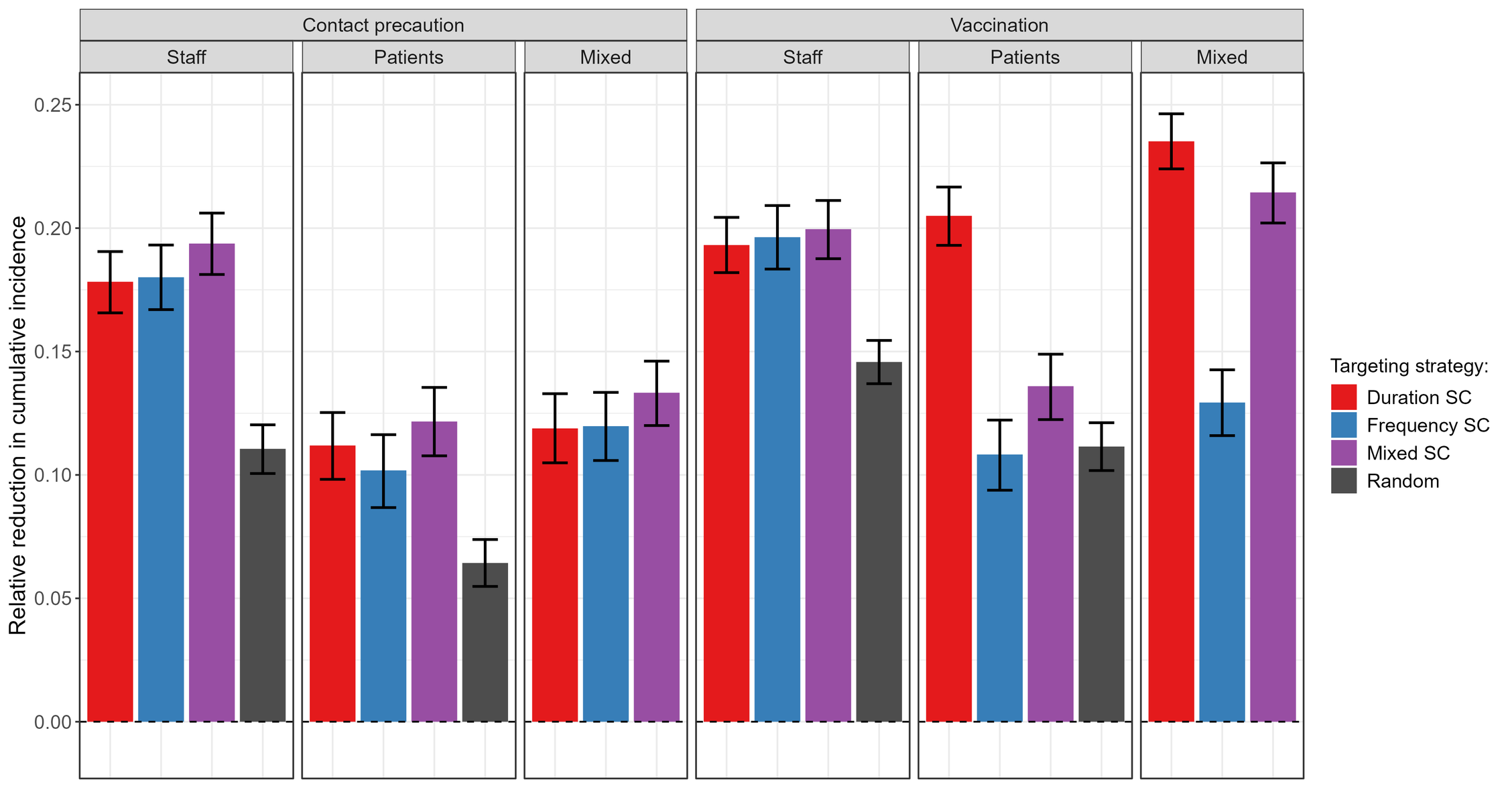

Supplement: S8 Fig — For each strategy, the bar indicates the median relative reduction in cumulative incidence, with 95% confidence interval, obtained for 500 simulations. (TIF) [file pmed.1004433.s008.tif]

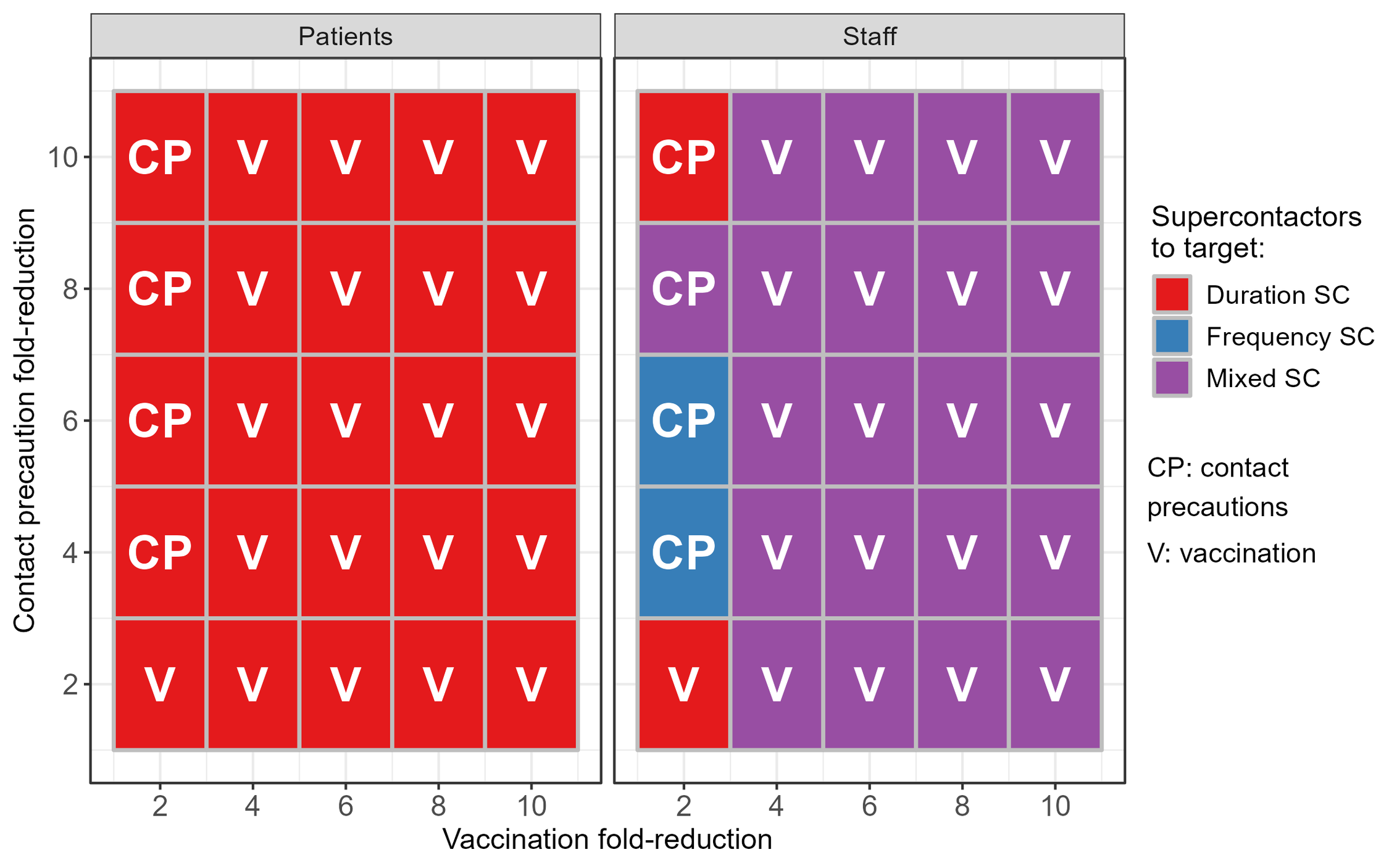

Supplement: S9 Fig — For each combination of assumed vaccine efficacy (x-axis) and contact precautions efficacy (y-axis), the colour of the square indicates which type of supercontactor should be targeted to achieve the highest reduction in MRSA colonisation, and the letter indicates which intervention should be used for this purpose. For example, if we are targeting staff (right panel) with either vaccination leading to a 4-fold reduction in transmission probabilities (x = 4) or contact precautions leading to a 10-fold reduction (y = 10), the best intervention is to vaccinate (V) a mix of duration- and frequency-based supercontactors (purple). (TIF) [file pmed.1004433.s009.tif]
