# Supplementary material for: Using contact network dynamics to implement efficient interventions against pathogen spread in hospital settings: A modelling study
Source: PLoS Med. 2024 Jul 30;21(7):e1004433. doi: 10.1371/journal.pmed.1004433 (PMC11341093; doi:10.1371/journal.pmed.1004433)
Supplement: S1 Text — (PDF) [file pmed.1004433.s010.pdf]

Using contact network dynamics to implement efficient interventions against pathogen spread in hospital settings: A modelling study

Quentin J. Leclerc[1,2,3,*,&], Audrey Duval[1,2,3,a#,&], Didier Guillemot[1,2,4], Lulla Opatowski[1,2,^], Laura Temime[3,5,^]

* quentin.leclerc@pasteur.fr

[&] these authors contributed equally

[^] these authors contributed equally

[1] Institut Pasteur, Université Paris Cité, Epidemiology and Modelling of Bacterial Escape to Antimicrobials (EMEA), Paris, France

[2] INSERM, Université Paris-Saclay, Université de Versailles St-Quentin-en-Yvelines, Team Echappement aux Anti-infectieux et Pharmacoépidémiologie U1018, CESP, Versailles, France

[3] Laboratoire Modélisation, Epidémiologie et Surveillance des Risques Sanitaires, Conservatoire National des Arts et Métiers, Paris, France

[4] AP-HP, Paris Saclay, Department of Public Health, Medical Information, Clinical research, Garches

[5] Institut Pasteur, Conservatoire National des Arts et Métiers, Unité PACRI, Paris, France

#a Current address : Imagine Institute, Data Science Platform, INSERM UMR 1163, Université de Paris, Paris, France

S1 TEXT – Detailed CTCmodeler description

Purpose and scope

CTCmodeler is composed of an individual-based model (IBM) previously described [1,2], and a module to compute model parameters. The aim of the model is to simulate pathogen transmission across the contact network between staff and patients in a healthcare setting. Here, it has been specifically tailored to examine the transmission of MRSA, leading to colonisation, in a long-term care facility recreating the conditions of the i-Bird study [3]. We then use the model to examine the potential impact of intervention strategies targeting different characteristics of the contact network to reduce the number of MRSA acquisition events over the period of interest. We aim to see if we can maximise this impact by identifying and targeting specific categories of individuals in the facility which we refer to as "supercontactors", who either have many contacts with different individuals, or spend the longest cumulative time in contact with others.

IBM description

The IBM module simulates the nosocomial transmission of a pathogen through an interindividual contact network. Three time schedules are used (i) time steps, (ii) days and (ii) weeks.

(i) The model runs using discrete 30-second time-steps. At each time step, the model simulates contacts and transmission events between individuals according to defined transmission probabilities between the groups these individuals belong to (patients or hospital staff). First, individuals in contact are identified, then if exactly one individual in the contact is colonised and one is susceptible, transmission is simulated stochastically to determine if the susceptible individual acquires the pathogen. When an individual newly acquires the pathogen, their status changes from "susceptible" to "colonized". A colonized individual will stay colonized for a duration sampled from a lognormal distribution at the time of the acquisition, after which their status reverts from "colonized" to "susceptible". This check to see if an individual has reached the end of their colonisation period occurs after the transmission steps.

(ii) Admissions and discharges operate every day. If an admission occurs, the probability that the new individual is admitted with a "colonized" status depends on the defined colonization

frequency at admission among patients. Swabbing, that will determine the observation process of colonization status, also operates daily, with a test for each individual to assess whether they are swabbed on that day.

(iii) Every week, the model selects the number of individuals that will be swabbed, and the corresponding swabbing days, according to a normal distribution informed by the average number of swabs taken for different staff or patient categories per day during the i-Bird study. The individuals are then chosen randomly amongst all those belonging to the corresponding category.

Outcomes of the model

To take into account a reporting process, the incidences and prevalence are those observed, and hence only include colonized individuals that have been swabbed. Sensitivity of the swab test is assumed to be perfect. The incidence is the number of weekly new acquisitions divided by the number of susceptible individuals the previous week.

Parameters estimation from data

Epidemiological parameters of the IBM are directly computed from the analysis of weekly longitudinal swab data obtained from the LTCF. Our model validation process is summarised in Fig A.

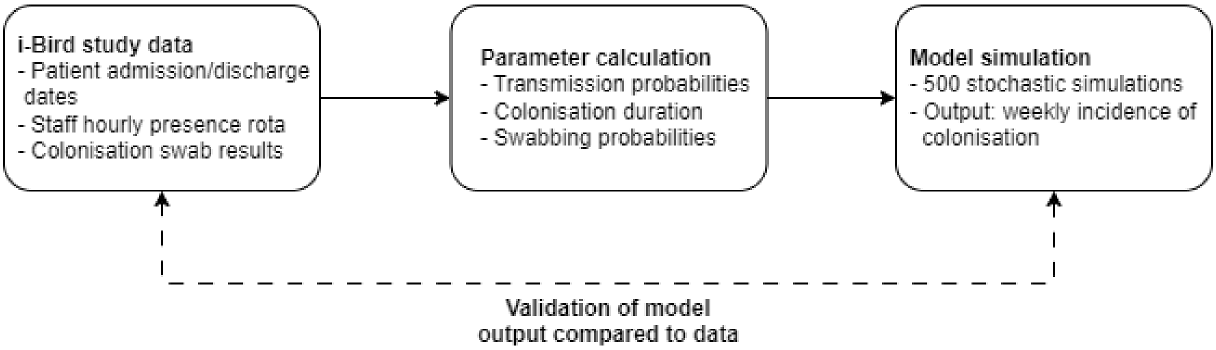

Fig A: Model validation process.

The following parameters are estimated:

Colonization duration

The mean and variance of the colonisation duration were computed separately for patients and hospital staff, using data from all the colonisation events recorded in the i-Bird data. The colonisation duration D_i of one colonisation episode *i* was estimated as:

$$D_i = \frac{D_{i,pos} + D_{i,neg}}{2} \quad (1)$$

Where $D_{i,pos}$ is the number of days between the first and the last positive swabs and $D_{i,neg}$ is the number of days between the last and the first negative swabs, respectively before and after those positive swabs. The colonisation duration D_i is therefore estimated as the average of these two durations, to account for uncertainty on the exact dates between the negative and positive swabs when the individual acquired/lost the pathogen.

For example, consider the following timeline for a colonisation episode: negative swab on day 5, positive swab on day 10, positive swab on day 32, negative swab on day 39.

The shortest possible colonisation duration would be $D_{i,pos}$ = 32-10 = 22 days. The longest possible colonisation duration would be $D_{i,neg}$ = 39-5 = 32 days. The average duration we record is therefore D_i = (32+22)/2 = 27 days.

Colonization probability at admission

The colonisation probability at admission was estimated for patients and hospital staff separately, by dividing the number of individuals with a positive swab within 48h of admission by the total number of individuals swabbed within 48h of admission.

Distribution of swabs across weekdays

The average number of swabs for each weekday *j* (i.e. Monday, Tuesday, Wednesday, Thursday, Friday) for each group of individuals *g* (i.e. patient or staff) are computed as:

$$P_{g,j} = \frac{1}{J_j} \sum_{w=1}^{W} R_{g,j}^{w} \quad (2)$$

Where J_j is the total number of specific days *j* (e.g. number of Mondays) over the investigation period, $R_{g,j}^{w}$ is the total number of individuals of group *g* swabbed on a day *j* on week *w*, and *W* is the total number of weeks. We assumed that there were no swabs taken on weekends, as observed during the i-Bird study.

Admission rate

The admission rates were estimated separately for each group (patient or staff), category (patient reason for hospitalisation or staff category) and ward, by dividing the total number of individuals of each group-category-ward combination admitted to the hospital by the length of the investigation period (84 days).

Length of stay

The mean and variance of the length of stay $Md_{g,c,s}$ for individuals of group g (i.e. staff or patient), category c (staff category or patient reason for hospitalization) and ward s are computed as:

$$E(Md_{g,c,s}) = \frac{1}{I_{g,c,s}} \sum_{i=1}^{I_{g,c,s}} \frac{1}{S_i} \sum_{j=1}^{S_i} D_j^i \tag{3}$$

$$Var(Md_{g,c,s}) = \frac{1}{I_{g,c,s} - 1} \sum_{i=1}^{I_{g,c,s}} \left[\frac{1}{S_i} \sum_{j=1}^{S_i} D_j^i - E(Md_{g,c,s}) \right]^2$$

Where $I_{g,c,s}$ is the total number of unique individuals of group g, category c and ward s across the study period, Si is the total number of hospital stays for the individual i and D_j^i is the duration of hospital stay j (in days).

Staff presence rate

Presence rate $TP_{h,c,s}$ of staff category c at hour h in ward s is computed as:

$$TP_{h,c,s} = \frac{\sum_{i=1}^{N^{cs}} \sum_{k=1}^{N^h} I_{i,k}}{N^h} \tag{4}$$

Where $I_{cr,i}$ is equal to 1 if the staff member i from category c allocated to ward s is present inside the hospital at instance k of the hour h and 0 otherwise. N^{cs} is the total number of staff of category c allocated to ward s. N^h is the number of instances of the hour h included in the study period (i.e. the number of days of the study period that contain the hour h).

Transmission probabilities

Transmission probabilities between two groups *g1* and *g2* (patients or staff) were estimated based on i-Bird contact data and swabs results as follows:

$$T_{g1\rightarrow g2} = \frac{1}{(N^w - n)} \sum_{w=n+1}^{N^w} \frac{\sum_{i=1}^{C^{g1}_{w-n\rightarrow w-1}} \sum_{j=1}^{S^{g2}_{w-n\rightarrow w-1}} I_{i,j,w-n\rightarrow w-1} \times A_{w,j}}{\sum_{l=w-n}^{w-1} D_{C^{g1}\rightarrow S^{g2},l}} \quad (5)$$

Using n=2 allows us to account for imperfect sensitivity of the swab tests, while n=1 assumes perfect sensitivity. We defined acquisition when one positive swab followed two previous negative swabs. N^w is the total number of weeks. For each week *w*, $C^{g1}{}_{w-n\rightarrow w-1}$ is the number of *g1* individuals with a positive colonisation status between *w-n* and *w-1* and $S^{g2}{}_{w-n\rightarrow w-1}$ is the number of *g2* individuals with a negative colonisation between *w-n* and *w-1*. $I_{i,j,w-n\rightarrow w-1}$ is equal 1 if the individual *i* (colonized) and the individual *j* (susceptible) during the period *w-n* to *w-1* were in contact, and 0 otherwise. $A_{w,j}$ is equal to 1 if *j* had an acquisition during week *w*, and 0 otherwise. $Dc_{t1\rightarrow t2,l}$ is the cumulative contact duration between colonized *g1* individuals and susceptible *g2* individuals during week *l*. The cumulative contact duration is obtained by summing all contacts between those individuals, with the duration of each single contact capped at one hour, to be consistent with the one-hour saturation in transmission probability which we used when simulating transmission. Only the first acquisition event of an individual was considered.

The resulting parameter values are listed in Table A. These values were all estimated using the i-Bird data directly. No other parameter values were required.

Table A. List of model parameters used for the agent-based model. NA: not applicable, for parameters not explored in the sensitivity analysis.

**Hospital characteristics**		
**Parameter**	**Baseline values in the model**	**Range explored for sensitivity**
Initial number of patients	151	NA
Initial number of hospital staff	236	NA
Number of swabs according to weekdays for patients (range of mean (range of variance))	4.45-61.36 (6.67-229.45)	NA

Number of swabs according to weekdays for hospital staff (range of mean (range of variance))	7.36-24.18 (4.56-102.56)	NA
**Pathogen characteristics**		
**Parameter**	**Baseline values in the model**	**Range explored for sensitivity**
Colonization frequency at admission among patients	19%	NA
Colonization frequency at start among hospital staff	37%	NA
Duration of carriage among patients (mean (variance)) in days	28.00 (755.01)	14.00 – 56.00
Duration of carriage among hospital staff (mean (variance)) in days	17.70 (270.59)	8.85 – 35.40
Patient to patient transmission probability per 30 seconds of contact	3.00×10^{-5}	$1.50 – 6.00 \times 10^{-5}$
Patient to staff transmission probability per 30 seconds of contact	1.19×10^{-4}	$0.59 – 2.38 \times 10^{-4}$
Staff to patient transmission probability per 30 seconds of contact	7.22×10^{-4}	$3.61 – 14.44 \times 10^{-4}$
Staff to staff transmission probability per 30 seconds of contact	2.51×10^{-4}	$1.25 – 5.02 \times 10^{-4}$

Simulations

Simulations ran over 84 days, with an initial 151 patients and 236 hospital staff members present, to reflect the duration and conditions of the data collection. For each scenario, 500 independent stochastic simulations were performed. Here, in order to replicate the i-Bird study conditions, the admission and discharge processes are deterministic and systematically correspond to the observed admission and discharged data. Transmission probabilities are fixed and calculated directly from the data, but the transmission process is stochastic; for each event, a number is drawn from a Uniform distribution between 0 and 1, and transmission only occurs if the number is smaller than the transmission probability. Colonisation durations are stochastic, and are drawn from a Lognormal distribution informed by the mean and variance of the durations observed during the i-Bird study. If a new patient or staff enters the facility for the first time, their colonisation status is determined by drawing a number from a Uniform distribution between 0 and 1; if it is lower than the colonisation frequency estimated from the data, the individual is colonised. The swabbing process is stochastic, with the number of swabs taken each day drawn from a Normal distribution parameterised using the mean and variance of the number of swabs per day observed during the i-Bird study. The identity of the swabbed individuals is determined stochastically, by selecting randomly individuals to swab from those present in the facility on that day.

The model was coded in C++ with the repast HPC 2.3.0 library, and is available in the following repository: https://gitlab.pasteur.fr/qleclerc/ctcmodeler. All simulations were performed on the Maestro cluster hosted by the Institut Pasteur. The R software was used to analyse the results and generate figures, with the corresponding code available in the following repository: https://github.com/qleclerc/ctcmodeler.

MInD-Healthcare Framework

The checklist of the MInD-Healthcare Framework is provided in Table B, with information on where each element can be found in the Supplementary Text and/or in the Main Text.

Table B. MInD-Healthcare Framework checklist.

Element	Checklist Item and Brief Definition	Reported in S1 Text section:
**1. Purpose and scope**	Purpose: Specify the primary problem under consideration or the objective(s) of the study. Scope: Specify the boundaries for which dynamics will included and which are ignored.	"Purpose and Scope"
**2. Entities, state variables, and scales**	Describe each entity, state variable, and scale. Entity: A distinct or separate object or actor that behaves as a unit; may interact with other entities or be affected by external environmental factors. Each entity's current state is characterized by its state variables. State variable: Attribute which performs at least 1 of the following functions: distinguishes an entity from other entities of the same type or category, or traces how the entity changes over time. Scale: Temporal and spatial resolutions and extents of the model.	"IBM description" (see also in Main Text: "Methods – Model description")
**3. Initialization**	Describe the initial states of the model entities and environment, (ie, at time t = 0) including how many entities of each type are present initially and the exact values of their state variables (or how they were set stochastically).	"Simulations"
**4. Process overview and scheduling**	Process overview: Specify who (ie, what entity) does what (ie, what actions are executed) and who is affected (ie, which entities and state variables). Scheduling: The order in which actions are taken and the order in which the effects of those actions are realized.	"IBM description"
**5. Input data**	Describe whether the model uses input from external sources such as data files or other models to represent processes that change over time.	"Parameters estimation from data"
**6. Agent interactions and organism transmission**	Agent interactions: Specify both direct interactions in which individuals encounter and affect others and any indirect interactions. Organism transmission: Describe how pathogen transmission is specified, including which interaction types result in changes to health states (eg, incident colonization, infection transmission).	"IBM description" (see also in Main Text: "Methods – Model description – Transmission process")

**7. Stochasticity**	Describe if stochasticity is part of the model and the major underlying reasons including which elements are modeled as fully stochastic, partly stochastic, or deterministic.	"Simulations"
**8. Submodels**	Describe the equations and algorithms used in the submodels. Include tables of parameter definitions, units, and values used.	"Parameters estimation from data"
**9. Model verification, calibration, and validation**	Describe the processes of verification, calibration, and validation. Verification: Process of ensuring the model was implemented correctly and meets the specifications of the model design. Calibration: Process of tuning the model parameters so that the model output matches a selected set of statistics from the real-world system or from simulated data. Validation: Process of evaluating how well the model represents the underlying truth of the real-world process it aims to represent.	"Parameters estimation from data" (see also in Main Text: "Results - Observed weekly MRSA incidence is well reproduced by simulations of network-based transmission")

References

1. Duval A, Smith D, Guillemot D, Opatowski L, Temime L. CTCmodeler: An Agent-Based Framework to Simulate Pathogen Transmission Along an Inter-individual Contact Network in a Hospital. In: Rodrigues JMF, Cardoso PJS, Monteiro J, Lam R, Krzhizhanovskaya VV, Lees MH, et al., editors. Computational Science – ICCS 2019. Springer International Publishing; 2019. pp. 477–487. doi:10.1007/978-3-030-22741-8_34

2. Smith DRM, Duval A, Pouwels KB, Guillemot D, Fernandes J, Huynh B-T, et al. Optimizing COVID-19 surveillance in long-term care facilities: a modelling study. BMC Medicine. 2020;18: 386. doi:10.1186/s12916-020-01866-6

3. Obadia T, Silhol R, Opatowski L, Temime L, Legrand J, Thiébaut ACM, et al. Detailed Contact Data and the Dissemination of Staphylococcus aureus in Hospitals. Salathé M, editor. PLoS Comput Biol. 2015;11: e1004170. doi:10.1371/journal.pcbi.1004170
