# Supplementary material for: Using contact network dynamics to implement efficient interventions against pathogen spread in hospital settings: A modelling study
Source: PLoS Med. 2024 Jul 30;21(7):e1004433. doi: 10.1371/journal.pmed.1004433 (PMC11341093; doi:10.1371/journal.pmed.1004433)
Supplement: Attachment — Submitted filename: review.pdf [file pmed.1004433.s011.pdf]

Dear Editorial team,

Thank you for the opportunity to submit a revised version of our article “Using contact network dynamics to implement efficient interventions against pathogen spread in hospital settings” to PLOS Medicine. We would also like to thank the reviewers for their deep reading of our work and constructive comments.

In response to these comments, we have modified the manuscript throughout which, we believe, has strongly clarified our methods and results, as well as the real-world applicability of our findings. Notably, we have introduced a new Supplementary Figure 1 alongside further text in Results and Methods to explain how each intervention affects the contact network. We have also added new paragraphs in the Introduction and Discussion to clarify the value of this work in the context of uncertainty surrounding the efficacy of these different interventions.

Following a reviewer comment which prompted us to further investigate why it was more effective to target different types of supercontactors for staff and patients, we identified two minor errors in the previous version of the analysis. First, the file which contained data on swabs collected during the i-Bird study missed negative swabs. Second, to make the analysis more realistic, we included a saturation of contact durations to 1h during the parameter estimation process to match the conditions of the subsequent simulations, as done in previous papers. The parameter values were updated following this change, resulting in slightly decreased colonisation durations, and increased staff-to-staff and patient-to-patient transmission probabilities (Supplementary Table 1). This modification did not affect the ability of the model to reproduce the observed incidence trends (Figure 1, updated with the correct swabs data). However, these parameter changes logically led to a slight change in the global conclusions on the best intervention to use. Since vaccination can reduce these transmission probabilities, while contact precautions can only reduce staff-to-patient and patient-to-staff probabilities (Supplementary Figures 1 and 5), the effectiveness of vaccination increased compared to contact precautions.

We have updated our results throughout the manuscript and, in response to this change and this reviewer comment, we have expanded the Discussion to further explain that the conclusion of which intervention is most efficient depends on the relative balance between within- and between-group transmission probabilities, since these interventions affect different parts of the contact network, and these distributions may strongly differ depending on structure and pathogen. We would like to thank again the reviewers for their insights. We believe these changes strongly improved the article and hope you will agree and consider this version suitable for publication in PLOS Medicine.

Best regards,

Quentin Leclerc, Audrey Duval, Didier Guillemot, Lulla Opatowski, Laura Temime

Editorial comments:

1) We think that your paper could offer a valuable contribution but as written it is not without limitations which we require you address prior to publication. Importantly when revising your manuscript please take care to ensure that it is presented in a manner that is accessible to the general reader as many of our readers will not be experts in infectious disease modelling. In particular, we found your figures difficult rather difficult to digest.

Thank you for the opportunity to submit a revised version of our manuscript. In response to reviewer comments, we have clarified several limitations surrounding our work and discussed how our conclusions are robust to these limitations. Please refer to our point-by-point responses to reviewer comments below for details.
In addition, we have updated several figures, either by removing some information and/or adding annotations to improve clarity (e.g. in Figure 2, we removed scenario numbers, and in Figure 3, we moved each legend label next to the corresponding line and removed a dashed line repeating a result from Figure 2).

2) We agree with the reviewers that better contextualisation of your findings in terms of real-world applicability would greatly improve the paper and that clearly defining the novel aspects of your study is required. In that context it would also be helpful to clearly define the long-term care facilities that you refer to – this can mean different things to different people in different parts of the world – much of the existing literature focusses on various hospital settings.

We have added details on the three interventions we modelled, clarified their mechanisms of action (lines 656-709), and added a new Supplementary Figure 1 to represent this. We have added a specific description of the long-term care facility in which the i-Bird data was collected at the beginning of the Results section (lines 109-115), and added a sentence in the Discussion to explain that the structure of LTCFs can vary between and even within countries.

3) We agree that the data pertaining to the hypothetical vaccine can be included in the main paper (PLOS Medicine has published many studies which model hypothetical vaccines) but as per the reviewers and the academic editor, please ensure that additional nuance is included.

We have clarified throughout that the vaccine we represent in the model is hypothetical.

4) Of all authors who submit modelling studies we ask that the following points are included in the main manuscript. Please review the list below, derived from Geoffrey P Garnett, Simon Cousens, Timothy B Hallett, Richard Steketee, Neff Walker. Mathematical models in the evaluation of health programmes. (2011) Lancet DOI:10.1016/S0140-6736(10)61505-X and ensure that each item is included in the relevant sub-sections of your manuscript:

* Please provide a diagram that shows the model structure, including how the disease natural history is represented, the process and determinants of disease acquisition, and how the putative intervention could affect the system.
We have added this as Supplementary Figure 1.

* Please provide a complete list of model parameters, including clear and precise descriptions of [the meaning of each parameter, together with the values or ranges for each, with justification or the primary source cited, and important caveats about the use of these values noted].

The list of model parameters was previously provided in Supplementary Table 1. We have added text alongside this table to state clearly that all parameters were directly estimated from the i-Bird study data.

*Please provide a clear statement about how the model was fitted to the data [including goodness-of-fit measure, the numerical algorithm used, which parameter varied, constraints imposed on parameter values, and starting conditions].
Model parameters were analytically calculated from the data collected during the i-Bird study rather than from fitting approaches such as MCMC. We have clarified this in Methods and Supplementary Text 1.

* For uncertainty analyses, please state the sources of uncertainties quantified and not quantified [can include parameter, data, and model structure].
Since the model parameters are directly calculated using i-Bird data, several uncertainties arise due to the i-Bird study design. We previously mentioned these limitations in the Discussion, but have expanded the text on this topic (lines 490-521).

* Please provide sensitivity analyses to identify which parameter values are most important in the model. Uncertainty estimates seek to derive a range of credible results on the basis of an exploration of the range of reasonable parameter values. The choice of method should be presented and justified.
We have clarified in Results, Discussion and Methods that we conducted a sensitivity analysis on transmission probabilities, carriage duration, and intervention effectiveness.

* Please discuss the scientific rationale for this choice of model structure and identify points where this choice could influence conclusions drawn. Please also describe the strength of the scientific basis underlying the key model assumptions.
We have clarified these points throughout, and we reference a previous model which relied on similar individual-based structures to quantify S. aureus dynamics across a contact network in a healthcare facility (DOI:10.1093/infdis/jis542).

Comments from the reviewers:

Reviewer #1: a) Could the authors elaborate on the 'simulated dynamic contact network' method used in the study. It would be beneficial for readers if this explanation is included either in the main manuscript or as an expanded section in the supplementary materials.
Complete details on this contact network can be found in [DOI:10.1101/2023.10.23.23296945], referenced in the main text. However, for improved clarity in this study, we have extended the section "Building synthetic contacts" in Methods to include a description of the contact network and how it was generated (lines 640-653).

b) The manuscript mentions the use of a log-normal distribution for the duration individuals remain colonized and a normal distribution for the scheduling of swabbing days. Could the authors provide a detailed rationale for choosing these specific distributions?
These distributions were chosen based on the distributions of observed colonisation durations and swabbing days during the i-Bird study. We have added a new Supplementary Figure 9 to show these distributions.

c) Regarding the equation for colonization duration that averages the days between the first and last positive swabs and the days around the last negative swab: the rationale for considering the entire period as colonization duration without division and the justification for halving the duration surrounding negative swabs warrant further clarification. It would be helpful if the authors could explain the underlying assumptions and their applicability more comprehensively.
*Thank you for highlighting this point. To clarify, the colonisation duration is computed as the average of a) the duration between the first <u>and</u> last positive swabs for an individual and b) the duration between the last negative swab **before** the first positive swab <u>and</u> the first negative swab **after** the last positive swab. This allows us to account for uncertainty around the exact dates when the individual acquired and lost the pathogen, since this may have occurred at any time between one negative and one positive swabs. We have rephrased the supplementary text to clarify this, and now provide an example to make the calculation clear.*

d) Does the model incorporate probabilistic elements, including distribution-based variations for all parameters, or does it solely rely on deterministic simulations? Clarification on the use of probabilistic methods versus deterministic assumptions in parameter modelling would provide valuable insight into the model's complexity and its ability to simulate real-world variability.
We have clarified in Methods (lines 614-627) and Supplementary Text that the model is stochastic, and added text to clarify how each stochastic process (transmission, colonisation duration etc…) functions.

e) The manuscript briefly discusses the impact of interventions such as re-allocation, contact precautions, and vaccination on model parameters, particularly highlighting a 6-fold reduction in transmission probability with vaccination implying an efficacy of 83%. Could the authors provide more extensive details on how these intervention effects were quantified and incorporated into the model? Specifically, the method for integrating the vaccination rate into the base-case scenario and its influence on model outcomes would be crucial for understanding the assumptions and implications of these interventions.
We have added a new Supplementary Figure 1 to illustrate how each intervention affects pathogen transmission across the contact network. In response to your comment and other reviewer suggestions, we have added further clarification in Introduction (lines 84-94) and Discussion (lines 506-521) that the efficacy of these different interventions is not necessarily known, hence the aim of our approach here to compare different possible efficacies, and identify intervention strategies which remain efficient across different scenarios. To further expand this point, we have added a new Supplementary Figure 8 to examine which type of supercontactor and which intervention is the most effective while varying the

efficacy of contact precautions and vaccination separately, as opposed to always assuming the same efficacy for both.

f) The manuscript would benefit from a more detailed presentation of the model validation and verification processes. While Figure 1 illustrates reproduced interactions, further elaboration on the methods and criteria used for model validation and verification would enhance the credibility and reliability of the model's findings.
We have added several sentences in the Results to justify our conclusion that the model reproduces the data based on overlap between estimates (lines 151-156). As mentioned in response to other reviewer comments, we have clarified throughout the text that all our parameters are directly calculated from the i-Bird data, rather than relying on a model-fitting approach such as MCMC. We believe that this methodological choice itself is a valid point to argue that our model is able to reproduce well the MRSA transmission dynamics across the LTCF.

Reviewer #2: Overall, I found this paper to be an enjoyable read, and an interesting one. I also commend the authors for making their code available. I do have a few potential areas where I believe the manuscript could use improvement:

1) The figures are very difficult to interpret in greyscale. More importantly, Figure 2 is nearly impossible to discern for several types of color blindness (using the CVSimulator app). I would suggest the authors consider one of several color blindness friendly pallets available in R.
Thank you for this suggestion, and for the recommendation of the CVSimulator app. For Figure 2, the use of colour is only aesthetic, since the information of which staff category is reallocated in each scenario can be derived from which squares are filled instead of white.
However, for Figure 3, we agree that our initial choice of colours did not help to clearly communicate our results. We have updated the colour palette to make sure that the two lines of interest in that graph (Healthcare assistants and Nurses) stand out from the other lines. We have also changed this figure to indicate the staff category reallocated next to the corresponding line for improved clarity.
For the other figures, we have confirmed with CVSimulator that the different colours (blue, red, grey, purple) can be distinguished.

2) The supplement does a good job describing the IBM, which is always a challenge. It would be nice if said description could be tailored to follow the MInD Framework, in hopes of making some progress in standardizing descriptions. https://urldefense.com/v3/__https://academic.oup.com/cid/article/71/9/2527/5802665__;!!JFdNOqOXpB6UZW0!sQfIBoeo89RzGlNgQfPCz2NQIXx8OCKHAI36riA_5PxN8OMOVQPRbU-Wh7Sn8z3I2_END-VBSyZnZ2xOLIFsZp-U$. I believe the supplement is not too far off from this already, and would represent relatively minimal effort.
In response to this and to an editorial comment, we have expanded the Supplementary Text to provide further information of model design, notably on the purpose and scope of the model as well as the stochasticity in the model simulations. We have added a new Supplementary Table 2 which contains the MInD framework checklist.

3) The "reallocation" intervention is somewhat opaque. The authors discuss how *allocation* works in the methods, and one can, I think, reverse engineer from there, but I'm not positive of what I'm looking at from both an algorithmic level and how it would translate into clinical policy. It might also be good to place your results in the context (cohere with or disagree with) of Mietchen et al.'s paper (https://urldefense.com/v3/__https://journals.plos.org/ploscompbiol/article?id=10.1371*journal.pcbi.1010352__;Lw!!JFdNOqOXpB6UZW0!sQfIBoeo89RzGlNgQfPCz2NQIXx8OCKHAI36riA_5PxN8OMOVQPRbU-Wh7Sn8z3I2_END-VBSyZnZ2xOLJWrlrIT$) looking at a compartmental model of staff assignment and MRSA in ICUs.
What we refer to as "staff reallocation" can be interpreted as a type of "staff cohorting". Here, we define reallocation as a change in the contact network to redistribute contacts amongst staff and patients whilst preserving healthcare needs. For example, if a patient should see a nurse once per day according to the observed contact network, in the "reallocation" contact network we maintain the constraint of one patient-nurse contact per day, whilst trying to always involve the same nurse in the contact whenever possible. We have clarified this definition in Results (lines 164-171) and Methods (lines 659-675).
On the other hand, "patient reallocation/cohorting" would refer to an intervention where patients are grouped by colonisation status, and staff are assigned either to the colonised or non-colonised group. In that case, staff would not act as vectors of transmission from colonised to non-colonised patients, as they would not be in contact with both groups. This is not the same as our "reallocation" intervention which does not rely on colonisation information, and instead aims to "optimise" staff-patient allocation to reduce the number of unique individuals in contact. Note that this approach would impact the spread

of any pathogen transmitted by close-proximity interactions across the network, not necessarily only MRSA.
Thank you for providing the reference to Mietchen et al, which does study a similar type of intervention than what we have used here and which we now reference in our manuscript (lines 402-405). This work found similar reductions in MRSA acquisition compared to our results (51.4% decrease by assigning staff to a fixed number of patients only).

4) It would be good if the authors engaged with the likelihood of a colonization blocking vaccine vs. one that reduces onward transmission or other vaccine mechanisms, especially in light of said vaccine being largely hypothetical at this point.
We have added a paragraph in the Discussion (lines 542-555) on potential vaccine impact based on available data. Here, what we represented in the model was a colonisation blocking vaccine, but we agree that other mechanisms could be possible. Our reason for choosing this mechanism was the interesting contrast we could then make with contact precautions, since while both interventions act by reducing transmission probabilities, the specific probabilities they affect are not the same (see the new Supplementary Figure 1 for details).

Reviewer #3: Thank you for giving me the opportunity to review this interesting manuscript. Leclerc and colleagues take advantage of the i-Bird study to parametrize an agent-based hospital model with the aim to find the best interventions to counteract the spread of MRSA in long-term care facilities. While the manuscript tackles an important and critical issue in hospital hygiene, it would in my view benefit a lot by pushing it from a modelling exercise to a clinically usable source of evidence.
I have no major issue with any technical part of the modelling study, but have the feeling that clinicians and hospital hygiene experts were not consulted well enough. The selection of potential interventions and the way they are reported follows technical aspects, and the different model components (starting with manipulation of agent-agent contacts and the resulting contact networks, going through manipulation of transmission probabilities, and ending with increasing the efficiency of transmission probability manipulations through network-based identification of the best targets) are well known and also a bit trivial. The study needs to shine by bringing this together with the real-world paramerization of the model and subject-matter knowledge of what is feasible.
Thank you for these comments. In response to this and other reviewer comments, we have clarified that our model parameters are directly calculated from the i-Bird data, as opposed to other approaches which rely on tools such as MCMC for model calibration. Our aim through this was to illustrate how modelling could be directly informed by data such as were collected during the i-Bird study, to then gain greater insight into the underlying transmission dynamics and the potential impact of interventions which would be clinically relevant. We have added further clarification in the Introduction (lines 84-94) and Discussion (lines 506-521) to acknowledge the uncertainty in intervention efficacy, and explain how we explore this uncertainty to identify conclusions on the most effective implementation strategy across a broad range of possible values.

Since there is currently no recommended vaccine against MRSA carriage or infection, I would argue that this can be shifted to the supplement. All parameters chosen for the potential vaccine are hypothetical anyway. It is a nice visualisation of what happens in a model when you manipulate something - but in my opinion this has currently no real-world correlate and thus also no benefit.
The assumptions made for the effect of interventions on transmission probability are broad - and of course affect the hierarchy of derived interventions. I would like the authors to decide - based on literature- what they assume to be the most appropriate parameter setting in each scenario - so that ranking by effectiveness and cost-effectiveness is possible. Something I think the manuscript needs; and I would like to see.
While there are currently no S. aureus vaccines available, this has been highlighted as a research priority due to the high burden of disease caused by this bacteria. Therefore, we believe there is value in trying to understand the potential benefit of such an intervention, and compare it to available alternatives to guide future implementation.
That said, we acknowledge the substantial uncertainty surrounding the potential effectiveness of all the interventions we simulate. Even for the more widely accepted intervention of improved contact precautions, previous studies have reported different efficacies to reduce pathogen transmission depending on setting-specific characteristics (compliance, products, care provided…). We previously mentioned this in our Introduction, and have added further context on this in the Discussion of our manuscript (lines 506-521 and lines 542-555). In addition, we have explored a range of possible efficacies for vaccination since, in the current context of the absence of such a vaccine, no data are available to inform this parameter.
Our aim in this work was therefore to acknowledge this uncertainty, but see if we could identify broad intervention strategies that would be efficient across a wide range of potential efficacies. We have clarified with the new Supplementary Figure 1 how each intervention affects transmission across the contact network. This representation highlights that while contact precautions and vaccination both act by reducing transmission probabilities, the specific probabilities they affect are not the same, resulting in an interesting comparison. Our conclusions then reflect which type of contacts are more

important with regards to transmission. We have rephrased our Introduction and Discussion to clarify this perspective and highlight the value of our approach.

While the platform designed can of course be adapted to other MDR bacteria, I think the way this is discussed in the manuscript is too optimistic. Most MDR bacteria have a relevant transmission component via fomites which is not covered in i-Bird and thus undermines the USP of this manuscript – the real-world parametrization. This would not be possible for VRE or MDR-Enterobacteriacaeae.
We agree that our model would not be applicable to all pathogens. As mentioned in the Discussion, this approach would only be valid for pathogens for which close-proximity interactions represent the primary, but not necessarily the only, route of transmission. For example, S. aureus transmission via fomites is possible, but we have previously shown that the contact network can explain most of the transmission observed during the i-Bird study [DOI:10.1371/journal.pcbi.1004170], and hence it is reasonable to assume that interventions affecting contacts would reduce transmission. Additional previous work in the context of the i-Bird study has shown that this was also the case for K. pneumoniae, but not E. coli [DOI:10.1371/journal.pcbi.1006496]. We previously mentioned this in the Discussion, but have now rephrased this section (lines 484-490) and added an additional sentence in Results (line 347) to make sure this point is clear.

Reviewer #4: The authors developed a detailed individual-based model to simulate MRSA transmission dynamics in a French long-term care facility. The study investigates at the impact of various interventions on MRSA colonization, differentiating between different types of interventions and different target populations. The authors created a contact network based on data collected from RFID sensor data recording close-proximity interactions from a previous study and were able to identify individuals that were influential in the contact network both in terms of frequency and the duration of the contact. The manuscript is generally well written and the dataset and the model itself are very detailed and as such interesting. However, I have concerns about the novelty of the results and relevance for public health. Most of the results in this study have been reported in the literature and so I would like to see more elaboration on how this study adds to known results. In addition, the authors use data from a single LTCF over a time period of only three months. This limits the generalizability of the results but also, it makes it difficult to fit the model to data over such a short time period. In particular, stochasticity of the data is hard to distinguish from In addition, I appreciate that several scenarios and sensitivity analyses were performed. However, the parameters for the scenarios seem to be arbitrarily picked and there is little explanation of how these parameters would translate to real-world scenarios in healthcare or whether there is existing evidence from the literature. The probably most surprising result was that the supercontactor to target differed between hospital staff and patients but the authors do not elaborate on these results nor provide further hypotheses for explanation. Generally, the Discussion section of the manuscript is more of a summary and repetition of the results from the study but I would like to see more of a "discussion" (I'll explain more in the line-by-line comments below):

Line-by-line comments:
Figure 1: 1b) shows the fit of the model to the weekly MRSA colonization data. I think this figure illustrates that over a short time period like this, there are a lot of stochastic effects even in the data (see the facet on Staff, second week of August). The prediction intervals of the model are very wide, sometimes ranging from 0 to 0.1. It's not clear to me that the model captures the dynamics well as stated by the authors. What are the measures by which the authors make that assessment? It seems that model fit was assessed only visually. To validate the model it would be useful to identify a measure by which model fit can be assessed more quantitatively (e.g. residual analysis or calibration plots) and provide these in the supplementary material.
Thank you for highlighting this. We have clarified that our parameters were all directly calculated from the i-Bird data rather than by fitting the model using an approach such as MCMC. We have added further text in Results to quantitatively describe our model's ability to reproduce the observed dynamics based on overlaps between the confidence and prediction intervals (lines 151-156).

The numbering of the scenarios in all figures (e.g. Figure 2) seem unintuitive and random to me. Could the authors add more explanation on how these were numbered and why they are not presented in consecutive order?
The numbering for scenarios was in progressive order based on which staff categories were targeted, not on efficacy. However, in light of this comment and an editorial comment, we have removed the numbering to make the figure easier to digest.

Figure 5 and 6: A 6-fold reduction in transmission probability in the main analysis seems quite high. It seems more likely that vaccination would have a smaller effect and maybe not even prevent acquisition of colonization but maybe only progression to clinical infection. What is the justification for using the 6-fold reduction in the main analysis?
We have now clarified throughout the manuscript that we focused on a hypothetical vaccine since no S. aureus vaccine is currently available. We agree that vaccines may have different effects, such as preventing only infection rather than colonisation, and we have added several sentences in the Discussion to clarify this (lines 542-555). We chose here to focus on prevention of colonisation since

such vaccines are being investigated for S. aureus (we have added references 50-52), and because this enabled an interesting comparison with the contact precautions intervention, which reduces different transmission probabilities (see new Supplementary Figure 1). Since this is a hypothetical vaccine, there is no estimate of efficacy available, so for comparability we considered an equal efficacy to contact precautions, which we assumed at 6-fold for Figure 5. However, we explored a wide range of possible efficacies from 2 to 10-fold to show that our conclusions on the value of targeting supercontactors remain valid across this range. We acknowledge that, in reality, the efficacy of vaccination and contact precautions may differ, and we have added the new Supplementary Figure 8 to highlight that our conclusions on the most effective implementation strategies are generally maintained, even in cases where the efficacies of the two interventions differ (see also Discussion lines 506-521).

Lines 342-344: Would it be possible to add more explanation to this?
We have added more discussion around our result that targeting a mix of frequency- and duration-based supercontactors is best for staff, but targeting duration-based supercontactors is best for patients (lines 319-328 and lines 422-428). Briefly, this depends on the relative values of the different transmission probabilities (staff-staff, patient-patient, patient-staff, staff-patient), and the relative amount of time spent by individuals with either individuals of the same group (e.g. staff with staff) or of the other group (e.g. staff with patients). We have added Supplementary Figures 5 to show this, and Supplementary Figure 8 to show that this is broadly consistent even if the efficacies of contact precautions and vaccination vary independently.

Line 444: While the contact network data is very detailed and a valuable source, it is from 2009. Could the authors comment on changes in these facilities and how it could affect the network and consequently their results?
We agree that hospital characteristics and contact networks can differ, either between facilities over by changes over time within a facility, and that this could affect our conclusions. We do not have specific information on the current structure of the facility in which the i-Bird study was conducted, but we have added further elements in the Discussion to clarify how the most efficient intervention strategy would depend on factors which may vary, notably the relative importance of transmission probabilities and of the amount of time spent by individuals in contact with either individuals of the same group (e.g. staff with staff) or a different group (e.g. staff with patients) (lines 319-328, lines 422-428 and lines 490-505).

Lines 502-508: The Reallocation intervention sounds similar to staff cohorting which I think is a more common used term. Did I understand that correctly? If yes, it could be helpful to mention that in the Methods section.
Thank your for pointing this out. In response to this and to another reviewer comment, we have added further explanations on this intervention in Results and Methods. Briefly, the "staff reallocation" intervention we implemented is a type of "staff cohorting", since it aims to decrease the number of unique contacts between patients and staff. For example, if a patient is scheduled to see a nurse everyday in the observed contact network, in the reallocation contact network we maintain this requirement of one patient-nurse contact per day, whilst trying to ensure that the patient always sees the same nurse for the entire period rather than a different nurse each day.
However, this is different from "patient reallocation/cohorting", generally considered as an intervention where patients are divided according to their colonisation status, and staff are assigned to exclusively take care of either non-colonised or colonised patients. Hence, staff would not act as vectors of transmission between colonised and non-colonised patients, since they would not interact with both groups. This is not how our intervention works here, since we do not separate individuals based on their colonisation status. We have made the distinction clear in the revised manuscript (lines 164-171 and lines 659-675).

Lines 509-522: The contact precaution scenarios assume reductions in transmission probability by 2,4,6,8,10 fold. What is the rationale behind these values? I think to see the application to public health, it would be useful to find literature on the impact of contact precautions (and their reinforcements) on the transmission probability. My main concern here is that these numbers might be too high and not realistic and thus it might not be a fair comparison to the other scenarios. Generally, in order to make scenarios between different intervention types comparable, it is important to consider how realistic these scenarios are.
We have clarified in the Introduction (lines 84-94) and Discussion (lines 506-521) that there is substantial uncertainty surrounding the efficacy of the interventions we investigate, with several new references to support this. This is why we examined several possible reductions in transmission probabilities, to assess if we could identify strategies which remained consistently effective across this range. However, we agree that the efficacy of each intervention may vary separately, which we did not examine in our initial analysis. We have added a new Supplementary Figure 8 to show that the best intervention implementation strategy generally remains the same even if we independently vary either contact precaution or vaccination efficacy.

Lines 523-531: The vaccination scenarios assume a reduction in acquisition probability of colonization. There's little (or no) literature on the effect of vaccines on S. aureus colonization. It seems that more evidence points towards a potential vaccine that reduces infection/disease severity. As such this scenario seems very hypothetical and probably unrealistic (even if they examine a range of parameters for the reduction in probability of colonization). If the authors still think that these are scenarios relevant for public health, I would like to see a discussion of why and literature that could support these assumptions.
We have clarified throughout the manuscript that we are examining the impact of a hypothetical vaccine. However, due to the high public health burden of S. aureus infections and the number of vaccines currently in clinical development, we believe it is relevant to examine the impact of a vaccine reducing colonisation. We have added references 50-52 which discuss vaccines against S. aureus colonisation. In addition, we have added some discussion surrounding vaccines against S. pneumoniae as examples of vaccines which reduce infection as well as colonisation, to illustrate that this type of protection is feasible in the context of bacterial vaccines (lines 542-555).

Comments from the Academic Editor:

I think this is an interesting study, and agree that we should offer the authors opportunity to respond to reviewers' comments (which are good).

I additionally recommend the authors consider:
1) the i-Bird contact data - these data were collected by proximity sensors, which I guess could indicate contact within a certain spatial distance (e.g. in the same room, but not in direct contact). But MRSA is predominately transmitted by direct contact. So, I think the authors should add to the limitations to discuss how their simulated contact networks may not reflect transmission dynamics, and might over, or underestimate transmission.
Thank you for this suggestion. The extent to which the contact network explains MRSA acquisition events in the i-Bird study has been evaluated in previous work [DOI:10.1371/journal.pcbi.1004170]. We have expanded our discussion on this point (lines 485-489), and also in response to comments by other reviewers on how well the contact network can explain the transmission of other pathogens.

2) Additionally, 1-2 sentences more in the Methods describing how i-Bird contact data were collected (how many sites, how many people, over what duration etc) would be helpful.
We have added this information at the beginning of the Results section, to better introduce the i-Bird study setting (lines 109-115).

3) I would like to see greater referencing to support the potential effectiveness of intervention scenarios to demonstrate that these are plausible effect assumptions.
We have added further discussion on the uncertainties surrounding the potential effectiveness of interventions, and the value of our modelling approach to explore these uncertainties (lines 506-521).

4) Similarly to the reviewer, greater nuance around the vaccination strategy is required, emphasising that this is a hypothetical vaccine throughout.
We have clarified throughout the manuscript that we examine a hypothetical vaccine.

5) The discussion should contextualise how the structure and function of healthcare institutions may vary from country-to-country, and speculate on what implications this would have for their results. (i.e. how generalisable are their findings?) This is particular important given the rather specialised nature of the setting where the i-Bird data were collected.
We have added further discussion on how hospital and pathogen characteristics, particularly transmission probabilities and the relative importance of contacts with individuals of the same group (e.g. staff-staff) or different groups (e.g. staff-patient) can impact our conclusions (lines 319-328, lines 422-428 and lines 490-521).
