# Supplementary material for: Using contact network dynamics to implement efficient interventions against pathogen spread in hospital settings: A modelling study
Source: PLoS Med. 2024 Jul 30;21(7):e1004433. doi: 10.1371/journal.pmed.1004433 (PMC11341093; doi:10.1371/journal.pmed.1004433)
Supplement: Attachment — Submitted filename: review2.docx [file pmed.1004433.s012.docx]

Requests from Editors:

COMMENTS FROM THE ACADEMIC EDITOR:

I have read through the revised manuscript and response to reviewers' comments. Overall, I think the authors have done well with their responses, and the revised manuscript is strengthened. I think the conclusions are now appropriately caveated regarding the hypothetical nature of the interventions.

*Many thanks for your positive comments on our manuscript.*

COMMENTS FROM THE EDITORS:

GENERAL

Thank you for your detailed responses to previous editor and reviewer comments. Please see below for further comments which we require that you address prior to publication.

Specifically referring to the methods and results sections, please reformat and organize your manuscript according to PLOS Medicine’s formatting requirements. Specific comments are detailed below and further guidance can be found here [https://urldefense.com/v3/__https://journals.plos.org/plosmedicine/s/submission-guidelines*loc-manuscript-organization__;Iw!!JFdNOqOXpB6UZW0!uLxKWuGK8Te6GqwrSZ0ZEA6PeoisInf5SnkUVC2y2Ou7g_QB4tnSmA28HJ9DSjzovaheFeT_Gzw5sY3rc_v7QPo2$](https://urldefense.com/v3/__https:/journals.plos.org/plosmedicine/s/submission-guidelines*loc-manuscript-organization__;Iw!!JFdNOqOXpB6UZW0!uLxKWuGK8Te6GqwrSZ0ZEA6PeoisInf5SnkUVC2y2Ou7g_QB4tnSmA28HJ9DSjzovaheFeT_Gzw5sY3rc_v7QPo2$)

Please note that many of the editorial requests detailed below pertain to specific formatting and content requirements. Some may have already been incorporated into the manuscript and some may not apply but please review the complete list of items and ensure that each item is included as necessary.

*Thank you for the opportunity to submit a revised version of our manuscript. Please find below our point-by-point responses to the further comments in italics.*

TITLE

Please revise your title according to PLOS Medicine's style. Your title must be nondeclarative and not a question. It should begin with main concept if possible. "Effect of" should be used only if causality can be inferred, i.e., for an RCT. Please place the study design ("A randomized controlled trial," "A retrospective study," "A modelling study," etc.) in the subtitle (i.e., after a colon).

*We have edited our title to “Using contact network dynamics to implement efficient interventions against pathogen spread in hospital settings: A modelling study” to align with PLOS Medicine’s style.*

DATA AVAILABILITY

Thank you for agreeing to make your code available which we appreciate. The Data Availability Statement (DAS) requires revision. As well as for the code, for each data source used in your study:

a) If the data are freely or publicly available, note this and state the location of the data: within the paper, in Supporting Information files, or in a public repository (include the DOI or accession number).

b) If the data are owned by a third party but freely available upon request, please note this and state the owner of the data set and contact information for data requests (web or email address). Note that a study author cannot be the contact person for the data.

c) If the data are not freely available, please describe briefly the ethical, legal, or contractual restriction that prevents you from sharing it. Please also include an appropriate contact (web or email address) for inquiries (again, this cannot be a study author).

*We have edited the Data Availability Statement to provide DOIs for our code repositories.*

STATISTICAL REPORTING

Throughout, including tables and figures, please quantify the main results with 95% CIs and p values.

When reporting p values please report as <0.001 and where higher as p=0.002, for example. If not reporting p values, for the purpose of transparent data reporting, please clearly state the reasons why not. When reporting 95% CIs please separate upper and lower bounds with commas instead of hyphens as the latter can be confused with reporting of negative values.

Please include the actual amounts and/or absolute risk(s) of relevant outcomes (including NNT or NNH where appropriate), not just relative risks or correlation coefficients. (example for absolute risks: PMID: 28399126).

*We have edited our reporting of 95% CIs throughout, clarified that all p values are <0.001 except where mentioned otherwise and, in response to a reviewer comment, we have clarified how these are calculated.*

LANGUAGE

Suggest caution with the language used to describe the different patient populations you study/simulate as ‘labels’ may cause offence. Please see below (methods and results) for more specific advice and please amend throughout all sub-sections of the manuscript and supporting files as necessary.

*We have made minor adjustments throughout the text to improve language clarity.*

ABSTRACT

Abstract Background: Please provide context of why the study is important. The final sentence should clearly state the study question.

Abstract Methods and Findings:

Please ensure that all numbers presented in the abstract are present and identical to numbers presented in the main manuscript text.

Please include the study design, population and setting, number of participants, years during which the study took place, length of follow up, and main outcome measures.

Please quantify the main results with 95% CIs and p values as detailed above. If not, to help facilitate transparent data reporting, please clearly state the reasons why not.

Please include any important dependent variables that are adjusted for in the analyses.

Please include the actual amounts and/or absolute risk(s) of relevant outcomes (including NNT or NNH where appropriate), not just relative risks or correlation coefficients. (example for absolute risks: PMID: 28399126).

In the last sentence of the Abstract Methods and Findings section, please describe the main limitation(s) of the study's methodology.

*We have edited the Abstract to include actual baseline colonisation incidence, 95% CIs, and the main limitation of our methodology.*

AUTHOR SUMMARY

At this stage, we ask that you include a short, non-technical Author Summary of your research to make findings accessible to a wide audience that includes both scientists and non-scientists. The authors summary should consist of 2-3 succinct bullet points under each of the following headings:

• Why Was This Study Done? Authors should reflect on what was known about the topic before the research was published and why the research was needed.

• What Did the Researchers Do and Find? Authors should briefly describe the study design that was used and the study’s major findings. Do include the headline numbers from the study, such as the sample size and key findings.

• What Do These Findings Mean? Authors should reflect on the new knowledge generated by the research and the implications for practice, research, policy, or public health. Authors should also consider how the interpretation of the study’s findings may be affected by the study limitations. In the final bullet point of ‘What Do These Findings Mean?’, please describe the main limitations of the study in non-technical language.

The Author Summary should immediately follow the Abstract in your revised manuscript. This text is subject to editorial change and should be distinct from the scientific abstract. Please see our author guidelines for more information: [https://urldefense.com/v3/__https://journals.plos.org/plosmedicine/s/revising-your-manuscript*loc-author-summary__;Iw!!JFdNOqOXpB6UZW0!uLxKWuGK8Te6GqwrSZ0ZEA6PeoisInf5SnkUVC2y2Ou7g_QB4tnSmA28HJ9DSjzovaheFeT_Gzw5sY3rcxZ-IZtE$](https://urldefense.com/v3/__https:/journals.plos.org/plosmedicine/s/revising-your-manuscript*loc-author-summary__;Iw!!JFdNOqOXpB6UZW0!uLxKWuGK8Te6GqwrSZ0ZEA6PeoisInf5SnkUVC2y2Ou7g_QB4tnSmA28HJ9DSjzovaheFeT_Gzw5sY3rcxZ-IZtE$)

*We have added an Author Summary in the revised article.*

INTRODUCTION

Please ensure that you address past research and explain the need for and potential importance of your study. Indicate whether your study is novel and how you determined that. If there has been a systematic review of the evidence related to your study (or you have conducted one), please refer to and reference that review and indicate whether it supports the need for your study.

METHODS and RESULTS

As above, thank you for the extensive amendments you have made which have substantially improved clarity.

Please move the ‘Methods’ sub-section to precede the ‘Results’ sub-section.

At times, the methodology is repeated and intertwined with the results – see lines 109 onwards as compared to lines 568 onwards, for example. The two subsections should exist as separate sub-sections (the methods preceding the results). Please amend throughout and to avoid repetition as much as possible.

*We have moved the “Methods” section, and revised the text throughout to avoid repetition.*

Line 109 – suggest ‘neurology’.

Line 109 onwards – as above, suggest caution when describing patient populations. Please refrain from referring to ‘neurologic…PVS…patients’, for example.

Line 110 – suggest ‘elderly care’.

Line 110 – suggest, ‘In addition, in-patients also included those in persistent vegetative state (PVS) and those in post operative recovery following general surgical and orthopaedic procedures.’ Or similar.

*We have revised the text according to your suggestions (please note that lines 109-110 are now lines 130-132).*

Line 113 – regarding the LCTF itself – is it designed for complex patients/procedures who/which are likely to require a long in-patient stay for either treatment or recovery? Additional nuanced details would be helpful for further context and to enable the reader to better appreciate transferability across settings.

*This LTCF is not designed for complex procedures, but rather for follow-up care and rehabilitation after procedures conducted elsewhere. We have clarified this in the description of the LTCF (lines 154-155).*

Please present numerators and denominators used to derive percentages.

Please provide the actual numbers of events for the outcomes, not just summary statistics or relative estimates.

*We have clarified that the baseline incidence of MRSA colonisation* *in the total population of 327 patients and 263 staff over the three-month period predicted by our model was 21 (prediction interval: 11, 31) for patients and 35 (prediction interval: 19, 54) for staff (lines 346-349). All relative reductions presented throughout can be converted by readers to actual amounts using this information.*

As above, where CIs are reported please also report p values. When a p value is given, please also include the test used to determine it.

*We have clarified in figure captions that CIs were derived using the Wilcoxon test to quantify whether results were significantly different from 0 (i.e. if the interventions have a significant impact or not). All p values are <0.001, unless otherwise specified.*

TABLES and FIGURES

Please ensure that each table/figure (including those in the supporting files) is affiliated to an appropriate title/caption/footnote which clearly descries the content without the need to refer to the text.

Please ensure that all abbreviations (including those used in statistical reporting) are clearly defined in the footnote(s).

Please indicate the meaning of all dots, lines and bars in the caption/footnote for the reader.

Figure 4 – please change the label ‘Geriatry’ on the axis to ‘Elderly care’.

We thank you for using a color palate suitable to those with color blindness.

*We have changed the label as requested.*

DISCUSSION

Please ensure that you present and organize the Discussion as follows: a short, clear summary of the article's findings; what the study adds to existing research and where and why the results may differ from previous research; strengths and limitations of the study; implications and next steps for research, clinical practice, and/or public policy; one-paragraph conclusion.

*We believe that our current Discussion contains the relevant information.*

REFERENCES

For in-text reference callouts please place citations in square parentheses separate by commas. For example, [1,3,6] or [1-3]. Please check and amend throughout all sub-sections of the manuscript and supporting files.

In the bibliography, please ensure that you list up to but no more than 6 author names followed by et al.

For all web references please ensure you include an, ‘Accessed [date].’

Journal name abbreviations should be those listed in the National Center for Biotechnology Information (NCBI) databases.

*We have checked that the references are aligned with those requirements.*

SUPPORTING INFORMATION

Please ensure to apply all guidance detailed above to the supporting information files.

Please cite your Supporting Information as outlined here: [https://urldefense.com/v3/__https://journals.plos.org/plosmedicine/s/supporting-information__;!!JFdNOqOXpB6UZW0!uLxKWuGK8Te6GqwrSZ0ZEA6PeoisInf5SnkUVC2y2Ou7g_QB4tnSmA28HJ9DSjzovaheFeT_Gzw5sY3rc6gC0gEZ$](https://urldefense.com/v3/__https:/journals.plos.org/plosmedicine/s/supporting-information__;!!JFdNOqOXpB6UZW0!uLxKWuGK8Te6GqwrSZ0ZEA6PeoisInf5SnkUVC2y2Ou7g_QB4tnSmA28HJ9DSjzovaheFeT_Gzw5sY3rc6gC0gEZ$)

In the published article, supporting information files are accessed only through a hyperlink attached to the captions. For this reason, you must list captions at the end of your manuscript file. You may include a caption within the supporting information file itself, as long as that caption is also provided in the manuscript file. Do not submit a separate caption file.

*We have added a list of captions at the end of our manuscript file.*

SOCIAL MEDIA

To help us extend the reach of your research, if not already done so, please detail any X (formerly Twitter) handles you wish to be included when we tweet this paper (including your own, your coauthors’, your institution, funder, or lab) in the manuscript submission form when you re-submit the manuscript.

Comments from Reviewers:

Reviewer #4: The authors have considerably improved their manuscript and addressed most of the points I raised adequately. I specifically commend the addition of figures in the supplement to visualize the differences between the interventions. I have a couple of more questions/comments:

*Many thanks for your positive comments on our manuscript.*

Figure 1: Does the legend from 1a) also apply to 1b) (that's what it seems like). Would it be possible to place the legend such that it is clear that it's for both figures?

*You are correct that the legend applies to both, we have now moved this legend to the middle of the figure to clarify this.*

Figure S8: This figure is helpful to compare the effects of the different interventions for different assumptions of the vaccine efficacy. However, it took me a while to understand the figure. Would it be possible to extend the figure (sub)title to explain how to read the figure?

*Thank you for your comment on this new figure. We have extended the figure caption to help with interpretation.*

Also it seems that for low vaccine efficacy (i.e. vaccination fold-reduction = 2) the most effective strategy is different than for higher vaccination fold-reductions. Is that a correct interpretation? Related to this, in the discussion (lines 505ff), the authors comment on this result by referring to the 2-fold reduction as extremely low. I think a 2-fold reduction could also be interpreted as high. Could the authors comment on the chosen range? The realistic range of values could be different for CP and vaccination, yet the authors chose the same range for both interventions. Based on the references for S. aureus and S. pneumo vaccination, what are the expected/observed vaccine efficacies?

*You interpretation is correct, since contact precautions appear to be the most effective intervention only if vaccine efficacy is low, which we mention on line 709. In reference 55 on S. pneumo vaccination, estimates for reductions in colonisation range up to 87%, although there is substantial variation depending on the reference population. The references on S. aureus vaccination do not provide vaccine efficacies. We assumed that a vaccine would only be approved if it had an efficacy greater than 50% (ie 2-fold reduction) to reduce the outcome of interest (this was the case for COVID-19 for example* [*https://www.who.int/news-room/feature-stories/detail/vaccine-efficacy-effectiveness-and-protection*](https://www.who.int/news-room/feature-stories/detail/vaccine-efficacy-effectiveness-and-protection)*), which is why we used this value as the lower limit of the range. We have used the same range for both interventions since our aim was to illustrate how these compared with similar characteristics.*

The authors refer to reference 50-52 for vaccines against S. aureus colonization. I think they mean 52-54? In reference 52, the S. aureus nasal colonization rates did not significantly change (if I understand it correctly). I wonder if that is an appropriate reference for what the authors are trying to show.

*Thank you for spotting this mistake, we have corrected the references. You are correct that reference 52 reports no significant impact of the candidate vaccine on S. aureus carriage, however our use of this reference here was to illustrate that research on this type of vaccine is ongoing. As we have highlighted in the article, we recognise that such a vaccine is currently not available.*

Figure S9: Similar to Figure S8, it would be helpful if the figure description could be extended. I assume these are probability distributions? How can the distribution for observed be so smooth?

*We have extended the figure caption. For ease of visualization we have chosen to represent the density of the distributions continuously, rather than discretely with histograms, which is why they appear smooth.*

Methods: It would be helpful if the authors could add in the main text how the 95% confidence intervals were calculated.

*We briefly mentioned at the end of the Methods that we used Wilcoxon tests to derive confidence intervals, but we have now clarified this in figure captions throughout.*
